# PET Neuroimaging: Insights on Dystonia and Tourette Syndrome and Potential Applications

**DOI:** 10.3389/fneur.2014.00183

**Published:** 2014-09-23

**Authors:** Pierpaolo Alongi, Leonardo Iaccarino, Daniela Perani

**Affiliations:** ^1^Department of Nuclear Medicine, San Raffaele Hospital, Milan, Italy; ^2^Bicocca University, Milan, Italy; ^3^Vita-Salute San Raffaele University, Milan, Italy

**Keywords:** PET, primary dystonia, Tourette syndrome, movement disorders, neuroimaging, statistical parametric mapping, treatment monitoring

## Abstract

Primary dystonia (pD) is a movement disorder characterized by sustained or intermittent muscle contractions causing abnormal, often repetitive, movements, postures, or both. Gilles de la Tourette syndrome (GTS) is a childhood-onset neuropsychiatric developmental disorder characterized by motor and phonic tics, which could progress to behavioral changes. GTS and obsessive–compulsive disorders are often seen in comorbidity, also suggesting that a possible overlap in the pathophysiological bases of these two conditions. PET techniques are of considerable value in detecting functional and molecular abnormalities *in vivo*, according to the adopted radioligands. For example, PET is the unique technique that allows *in vivo* investigation of neurotransmitter systems, providing evidence of changes in GTS or pD. For example, presynaptic and post-synaptic dopaminergic studies with PET have shown alterations compatible with dysfunction or loss of D_2_-receptors bearing neurons, increased synaptic dopamine levels, or both. Measures of cerebral glucose metabolism with ^18^F-fluorodeoxyglucose PET (^18^F-FDG PET) are very sensitive in showing brain functional alterations as well. ^18^F-FDG PET data have shown metabolic changes within the cortico-striato-pallido-thalamo-cortical and cerebello-thalamo-cortical networks, revealing possible involvement of brain circuits not limited to basal ganglia in pD and GTS. The aim of this work is to overview PET consistent neuroimaging literature on pD and GTS that has provided functional and molecular knowledge of the underlying neural dysfunction. Furthermore, we suggest potential applications of these techniques in monitoring treatments.

## Introduction

The dystonias are a heterogeneous group of hyperkinetic movement disorders characterized by disabling spasms of the body due to sustained or intermittent muscle contractions causing abnormal, often repetitive, movements, postures, or both (1).

There has been a rationalization of the classification of dystonia and a greater understanding of the causes of dystonic movements from the study of genetics, neurophysiology, and functional imaging in the most prevalent form of dystonia syndrome, primary dystonia (pD) (2). Three basic parallel approaches are used to classify pD: age of onset (early or late), distribution of affected body parts (focal, segmental, or multifocal), and cause (presence or absence of genetic factors) (2).

Gilles de la Tourette syndrome (GTS) is a childhood-onset complex neurobehavioral disorder defined by motor and phonic tics, which can be often complicated by comorbid conditions that could progress to behavioral changes (3, 4). GTS and obsessive–compulsive disorders (OCD) indeed are often seen in comorbidity, also suggesting that a possible overlap in the pathophysiological bases of these two conditions. The exact etiology of GTS remains unknown, but the impairment of cortico-striatal-thalamo-cortical (CSTC) network seems to be the primary site of underlying damage.

PET molecular and functional neuroimaging techniques have been used to investigate the neural basis of movement disorders, as well as to identify potential cerebral targets for medical and surgical therapies. These techniques have provided evidence for specific alterations of both glucose metabolism and neurotransmitter systems in movement disorders, also useful for differential diagnosis (5). A very intriguing and promising application of ^18^F-fluorodeoxyglucose PET (^18^F-FDG PET or perfusion ^15^O-H_2_O) is the monitoring of the brain stimulation treatments in movement disorders (i.e., deep brain stimulation – DBS), together with the related assessment of the functional metabolic post-stimulation changes. Also, statistical parametric mapping (SPM) procedures allow robust voxel-based single-subject analysis (6) useful for treatment monitoring. The chance to run voxel-based single-subject analyses is of particular interest, given the low number of patients usually undergoing surgical stimulation treatments. To date, multiple reports have used similar approaches to define how stimulation treatments exert their effects in various movement disorders, like in pD (7, 8), and GTS with PET molecular imaging (9). Since the clinical manifestations of these movement disorders may be very heterogeneous and peculiar, PET monitoring could play a supportive and crucial role in evaluating the effects of the treatments and the progression of the diseases.

Functional (fMRI) and structural magnetic resonance imaging using advanced techniques as diffusion tensor imaging (DTI) have also been applied to the study of brain functional mechanisms and macrostructural abnormalities underlying movement disorders. As for pD, in sporadic cases as well as in genetic mutation carriers, DTI showed changes in the integrity of white matter fiber tracts by means of indices like fractional anisotropy or mean diffusivity (10). Similarly, DTI-based studies in GTS yielded clear evidence of reduced microstructural integrity of white matter extending beyond motor pathways. These studies suggest that alterations of the connecting systems in these diseases, also with evidence for anatomo-clinical correlations (11, 12).

Advances in PET medical technology imaging have helped to further clarify the pathophysiology of specific movement disorders, showing the associated metabolic and molecular alterations. In pD, ^18^F-FDG PET revealed a consistent pattern of hypermetabolism, encompassing basal ganglia, and sensorimotor pathways (5, 13). In GTS, ^18^F-FDG PET provided evidence for hypermetabolism occurring at sensorimotor cortex level and hypometabolism in the limbic cortex and striatum (14). In addition, cerebral perfusion activation studies in GTS (by means of ^15^O-H_2_O and PET) have also provided functional evidence for activations in the cerebellum, insula, thalamus, and putamen during tic release. This prominent involvement of cerebellum and insula, suggested that their recruitment in tic initiation and execution (15).

In addition, PET molecular imaging, with specific radiotracers, such as [^11^C]raclopride, alpha-[^11^C]methyl-l-tryptophan (^11^C-AMT), ^11^C-flumazenil, ^11^C-WIN (DA transporter antagonist), ^11^C-MCN (a 5-HT_2A_-R antagonist), and ^11^C-MDL (a SERotonine Transporter – SERT – antagonist) have represented a unique tool for *in vivo* evaluation of the biochemical mechanisms underlying motor dysfunctions. Studies of the neurotransmitters pathophysiology revealed multiple and complex underlying biochemical disorders.

All above supports the crucial role of PET investigations in better identifying the underlying pathophysiological mechanisms of pD and GTS disorders, showing also a potential application in treatment monitoring. The main advantage of such techniques is the possibility to *in vivo* investigate the changes in brain metabolism and neurotransmission systems before and after treatment.

This brief overview addresses the functional alterations in the networks and the interactions with neurotransmission systems in pD and GTS. It also discusses the innovative use of PET molecular imaging as a tool for monitoring interventional therapy and its use as an outcome measure.

## Primary Dystonia

### Clinical characterization

Primary dystonia typically begins in late childhood or adolescence and it is traditionally attributed to basal ganglia dysfunction (16). It is of note that no specific pathological lesions of these structures have been consistently evidenced in post-mortem studies (17). pD is defined as a movement disorder characterized by sustained or intermittent muscle contractions causing abnormal, often repetitive, movements, postures, or both (1). Dystonic movements are typically patterned and twisting, and may be tremulous too. These movements are often primed or worsened by voluntary actions and associated with overflowing muscle activation. pD can be classified along two axes, as defined by Albanese et al. (18): (1) clinical characteristics including age at onset, body distribution, temporal pattern, and associated features (additional movement disorders or neurological features); and (2) etiology and inheritance. The clinical characteristics fall into several specific dystonia syndromes that might help in a better diagnosis and strategic treatment. In regards to genetic forms, DYT1 and DYT6 are the most common and are inherited as autosomal dominant traits with incomplete penetrance (19–21).

### ^18^F-FDG PET evidence

Several ^18^F-FDG PET neuroimaging studies have provided knowledge on the functional anatomy of pD. Lehericy et al. (22, 23), in their comprehensive reviews on neuroimaging of dystonia using fMRI and ^18^F-FDG PET, highlighted the hyperactivity of premotor and prefrontal areas and the hypoactivity of primary sensorimotor areas. Thus, since regions other than the basal ganglia are involved in dystonic movements, neuroimaging evidence supports the hypothesis of pD as a circuit disorder. Coherently, most of the literature converges in supporting the involvement of both basal ganglia-thalamo-cortical and cerebello-thalamo-cortical pathways (19, 23, 24).

More specifically, ^18^F-FDG PET has been used in different dystonic disorders including primary generalized dystonia and DOPA-responsive dystonia (DRD), as well as in focal dystonic syndromes such as spasmodic torticollis, writer’s cramp, and blepharospasm (25–27). Common findings concern functional metabolic abnormalities in the basal ganglia and associated outflow pathways to sensorimotor cortex and to other regions involved with motor control (5). Hutchinson et al. studied the brain metabolic pattern in six subjects with essential blepharospasm compared to normal volunteers. They showed that the clinical manifestations were associated with abnormal metabolic activity in the pons and cerebellum, and with additional abnormalities also in cortical eyelid control regions (27).

Asanuma et al. (10), by studying subjects’ torsion dystonia-related pattern, showed a relative increase in the metabolic activity of the posterior putamen, globus pallidus (GP), cerebellum, and supplementary motor area (SMA).

All these results contribute to consider pD as a neurocircuit disorder, involving the cortico-striato-pallido-thalamo-cortical and cerebello-thalamo-cortical pathways, which are recognized as a cause of the clinical manifestations (28, 29). An aberrant motor response is thought to result from abnormal processing at a level of central sensorimotor integration and as a disturbance of sensory input at the level of spinal interneuronal circuits (23).

^18^F-fluorodeoxyglucose PET imaging applied to manifesting and clinically non-manifesting gene carriers has offered the possibility of identifying alterations in circuit functional connectivity associated with both genotype and penetrance. For example, in regards to torsion dystonia, some authors hypothesized that its related metabolic pattern (TDRP, hypermetabolic at putamen, pallidus, SMA, and cerebellum levels) can potentially be used as a marker in linkage studies to identify potential gene carriers among family members of pD patients (28). Similarly, in patients with pD due to DYT1 mutation, an abnormal metabolic brain networks was reported, characterized by hypermetabolism in the basal ganglia, SMA, and cerebellum (10). Trost et al. have quantified the metabolic activity of this network in patients carrying different pD mutations, in order to investigate whether this functional abnormality is linked to genotype. Their findings suggest that a consistent abnormal metabolic topography that is not genotype specific, being present in carriers of other pD mutations (30).

Recently, Carbon et al. (31) identified that brain regions with metabolic changes in DYT11 myoclonus-dystonia (DYT11-MD) showed specific patterns of metabolic abnormalities, involving connecting pathways between the cortex, basal ganglia, thalamus, and cerebellum. In addition, they compared metabolic abnormalities in DYT11-MD with those found in other forms of hereditary dystonia and in post-hypoxic myoclonus. They found significant DYT11 genotype-specific metabolic increases in the inferior pons and in the posterior thalamus as well as reductions in the ventromedial prefrontal cortex. Significant phenotype-related increases were also present in the parasagittal cerebellum. This latter abnormality was shared with post-hypoxic myoclonus, but not with other forms of pD. These findings were consistent with the hypothesis of a sub-cortical myoclonus generator presence in DYT11-MD, particularly likely to involve the cerebellum (31). This evidence shows how ^18^F-FDG PET imaging can help identify different abnormal metabolic networks in specific variants of movement disorders such as in the context of pD.

### PET neurotransmission studies

Primary dystonia is a very complex disease spectrum, in which a pathological phenomena elicits a cascade of events encompassing different networks and molecular systems, from dopamine to GABA (32, 33) and also, as recently shown, acetylcholine (ACh) (34). In pD, PET and appropriate radiotracers have been used, in particular, for the assessment of dopaminergic system to investigate whether alterations in striatal receptor binding are showed by patients. Historically (1997), landmark evidence showed alterations of D_2_-R binding in putamen in focal dystonias, as measured by ^18^F-spiperone (reduced by up to 29%) (35). More recently, the adoption of ^11^C-raclopride (a very specific radioligand for D_2_-R) yielded consistent results across various studies in different forms of pD. Künig et al. (36) evaluated in 14 patients with DRD, the dopamine D_2_-R binding by ^11^C-raclopride PET in comparison with 16 levodopa-treated Parkinson’s disease (PD) patients and 26 healthy controls (HCs). The DRD patients showed increased ^11^C-raclopride binding in the putamen and caudate nucleus when compared with both controls and PD patients. The results were interpreted as reflecting reduced tracer displacement by endogenous dopamine, or as an alteration of the receptor’s features due to chronic dopamine deficiency. In addition, the differences in ^11^C-raclopride binding between DRD and PD patients in the caudate nucleus, suggest that this structure may be of pathophysiological relevance in the presentation of the clinical features of both diseases (36). Coherently, Rinne and co-workers investigated the integrity of striatal dopaminergic system in seven patients with DRD using PET with different radiotracers to evaluate dopamine transporter functioning (DAT ligand ^11^C-CFT), D_1_ (^11^C-NNC756), and D_2_-R (^11^C-raclopride). The results showed increased striatal dopamine D_2_-R availability in DRD with unchanged dopamine D_1_ receptors and DAT ligand binding. The increased D_2_-R availability seems to be due to reduced competition by endogenous dopamine or a compensatory response to dopamine deficiency, or both (37). ^11^C-raclopride has been also recently used to investigate another focal dystonia, namely, writer’s cramp (38). Berman et al. analyzed striatal D_2_/D_3_ availability at rest and during endogenous dopamine release during sequential finger tapping and speech production tasks in 15 patients with writer’s cramp and 15 matched HC subjects. This work showed that patients with writer’s cramp may have divergent patterns of striatal dopamine release during both a motor task (involving the dystonic hand) and an unrelated asymptomatic task, like sentence production (38). On the other hand, ^11^C-raclopride PET showed its efficacy also in investigating commonalities and divergences between different forms of genetic mutation dystonia, like DYT1 and DYT6 (33, 39).

Asanuma et al. (39) studying the DYT1 mutation with ^11^C-raclopride, found a 15% reduction of tracer binding in caudate and putamen in subjects without clinical manifestations. While this could have been interpreted as a trait feature of DYT1 mutation, later works with the same radiotracer showed similar reductions a in DYT6 mutation as well (33). These changes, which may be present in different degrees in the DYT1 and DYT6 genotypes, are likely to represent susceptibility factors for the development of clinical manifestations in mutation carriers (33). It is of note that a recent study revealed unaltered D_1_ receptor binding (by means of ^11^C-NNC112 PET) in primary focal dystonias when compared with HCs (40).

This suggests that dopaminergic post-synaptic alteration may be an early pathological trait of the condition, not sufficient *per se* in eliciting the full clinical phenotype (5).

Since preclinical and indirect clinical evidence suggest that molecular changes also of the GABAergic control system might represent a key dysfunctional component leading to disinhibition of the sensorimotor system (41, 42). Garibotto et al. used ^11^C-flumazenil PET in patients with sporadic and DYT1 mutation pD in order to assess the integrity of the GABAergic system. The results revealed a reduction in GABA_A_ receptor expression or affinity, both in DYT1 carriers and sporadic patients in primary motor and premotor cortex, primary and secondary somatosensory cortex, and in the motor component of the cingulate gyrus. Clearly, a deficit in GABAergic function might indeed result in abnormalities of neuronal inhibition affecting both the motor and somatosensory systems. In particular, the reduced inhibitory control in somatosensory cortices might suggest that the GABA system plays a crucial role in the modulation of the afferent signal to the somatosensory cortex in pD (43). Noteworthy, a MR spectroscopy study in patients with pD, revealed a significant decrease in GABA levels in the sensorimotor cortex and lentiform nuclei contralateral to the affected side, thus, providing indirect evidence supporting the relevant role of this system in pD (44).

As claimed by Tanabe and co-workers, even-though anti-cholinergic medications are effective in DYT1 and other forms of dystonia, this does not necessarily imply a primary role of ACh in these disorders. In point of fact, the abnormal cholinergic functioning may result as a secondary effect of the altered dopaminergic neurotransmission in the striatum (34) and this imbalance may have a role in symptom generation, as showed recently in DYT1 animal models (45).

## Gilles de la Tourette Syndrome

### Clinical characterization

Gilles de la Tourette syndrome is a childhood-onset neuropsychiatric developmental disorder characterized by motor and phonic tics that are defined as involuntary or semivoluntary, sudden, intermittent, repetitive movements (motor tics), or vocalizations (phonic tics) (3). Comorbidities are very common, in particular, OCD and attention deficit hyperactivity disorder (46). It is of note that the exact etiology of GTS remains unknown. Volumetric MRI in GTS provided evidence for correlations between tic severities and volume of specific structures [e.g., caudate, see Ref. (47)] and also for abnormal gray matter volumes in prefrontal cortex in children and adults [see Ref. (48, 49), and for review see Ref. (50, 51)]. Functional neuroimaging techniques, such as single-photon emission computed tomography (SPECT), PET, and fMRI have provided some evidence for the underlying pathological mechanisms in GTS that enabled new hypotheses on its pathophysiology to be formulated (50, 51). In particular, these studies suggest that the involvement of the CSTC network in the pathophysiology of tics and associated psychopathological manifestations in GTS (19).

### ^18^F-FDG PET evidence

^18^F-fluorodeoxyglucose PET investigations, also through *ad hoc* parametric measurements and/or voxel-wise statistical analysis, have shown that regions other than the basal ganglia circuits may be involved in GTS [see for example Ref. (14, 52)]. It has been hypothesized that abnormal connections between basal ganglia, thalamus, and cortex (within the CSTC circuitry) may be specifically associated to this condition (53). GTS has been significantly characterized by (a) lower metabolic rates in caudate nucleus and thalamus, (b) possible association with hypoactivity in lentiform nuclei and hippocampal formation, and (c) higher metabolic rates in the sensorimotor cortices (14, 15, 54, 55).

Pourfar et al. showed resting-state reduced metabolic activity in the striatum and orbitofrontal cortex, and it was associated with relative metabolic increases in premotor cortex and in cerebellum in GTS-related metabolic pattern. Further analysis of the same cohort, revealed that OCD symptoms in GTS patients were related with a second metabolic pattern, characterized by (a) reduced metabolic activity in the anterior cingulate and dorsolateral prefrontal cortices and (b) associated increases in primary motor cortices and precuneus. The authors conclude that the different clinical manifestations of GTS are associated with the expression of these two distinct abnormal metabolic brain networks (14).

Correlation analysis between glucose metabolism and clinical evidence contributed in characterizing the system networks affected in this neuropsychiatric disease, which might be useful in correct identification of the disorder. ^18^F-FDG PET studies have shown significant correlations between the presence of tics and hypermetabolism in several brain regions, including the medium and lateral premotor cortices; the primary motor cortices; the inferior parietal cortices; as well as the anterior cingulate cortex, putamen, and caudate and Broca’s area (53). These results support hyperactivity of the systems involved in motor planning/structuring and in the processing of sensory inputs at the basis of GTS symptoms. In summary, GTS shows heterogeneity of cortical and sub-cortical metabolic alterations depending on different factors not yet completely identified, thus, hindering the characterization of a specific metabolic pattern. Nonetheless, ^18^F-FDG PET at single-subject level represents a useful tool for the assessment of brain functional deficits, giving the chance to monitor personalized brain target therapy and to assess the efficacy of treatments.

### PET neurotransmission studies

Few PET molecular studies of neurotransmission systems provided insights on the underlying pathophysiology of GTS. Historically, GTS has always been linked to a dysregulation of dopamine neurotransmission system (52, 56), also given the evidence for reductions of TIC severity in treatments with D_2_-R antagonists (57). Coherently, early post-mortem studies also provided evidence for alterations in second messenger system (58) and also for a possible relation between clinical phenotypes of GTS and dopamine innervation in the striatum (^3^H-mazindol showed an increased density of uptake sites) (59). To date, other neurotransmission systems like serotonin or GABA have been investigated in GTS (15, 60, 61).

Singer et al. tested the presynaptic dopamine release from the striatum after amphetamine administration in GTS adult patients using ^11^C-raclopride PET. Results were consistent with the possibility that the pathologic mechanisms in GTS relate to an abnormal regulation of the phasic dopamine response, resulting in hyper responsive spike-dependent dopaminergic system activation. Thus, a tonic/phasic imbalance in the DA system may help to explain the DA pathophysiology associated with GTS that could be crucial in developing potential pharmacologic treatments (62). Behen et al. used ^11^C-AMT PET to assess global and focal brain abnormalities of tryptophan metabolism and their relationship to behavioral phenotype in children with GTS and healthy age-matched controls. Their results show cortical and sub-cortical abnormalities of tryptophan metabolism in GTS, in particular, in the dorsolateral prefrontal cortex (DLPFC) and bilateral thalamus levels, providing a strong neuroimaging evidence for a role of serotoninergic mechanisms in the pathophysiology of GTS (60). Saporta et al. (61), using ^11^C-AMT PET and DTI investigated both structural white matter abnormalities and serotonin synthesis in children with GTS. The authors hypothesized that microstructural alterations and related altered connectivity in CSTC might have been the primary abnormality in GTS, then inducing altered neurotransmission. They found an asymmetric immature microstructure in the caudate nucleus that was associated with abnormally increased serotonin synthesis. The authors suggest that the increased serotonin synthesis in the caudate could be due to cortical disinhibition, in the context of abnormal cortico-striatal connectivity. On this basis the authors suggest serotonin system as a possible new therapeutic target in GTS (61). Interestingly, Lerner et al. (63), in the first study of GABA neurotransmission system in GTS patients using ^11^C-Flumazenil and PET found a consistent decrease of GABA_a_ receptors in multiple limbic regions such as amygdala, ventral striatum, thalamus, and also at the insula level. An increase of these receptors was also present in the cerebellum, in the bilateral substantia nigra (SN) at periaqueductal gray (PEG) level and in the right posterior cingulate cortex (63).

In addition, besides the evaluation of isolated systems, insights on underlying GTS pathophysiology have been provided by studies on neurotransmitters inter-modulation. Wong et al. (64) studied both dopaminergic and serotoninergic neurotransmitter systems at transporter and at receptor density levels, as measured by, respectively, ^11^C-raclopride and ^11^C-WIN (Dopamine Active Transporter – DAT antagonist) or ^11^C-McN (5-HT_2a_R antagonist) and ^11^C-MDL (SERT antagonist). In particular, they tested whether there was a connection between phasic DA release (DA_REL_) induced by administration of amphetamine and levels of 5-HT in patients with GTS plus OCD comorbidity. They found a strong correlation between DA_REL_ and low levels of 5-HT, suggesting that “DA_REL_ is a primary defect in GTS” (64).

Further investigations in larger patient groups are necessary in order to further elucidate the complex neuromodulation changes in GTS.

## PET Neuroimaging and Stimulation Treatment Monitoring: Evidence in pD and GTS

As anticipated in the introduction, a promising approach is to use PET techniques (mainly perfusion ^15^O-H_2_O and/or ^18^F-FDG PET) to monitor therapy efficacy in a variety of conditions and across different type of medical and surgical treatments. For example, PET techniques have shown a high value in monitoring the effects of surgical therapies like DBS, which has been extensively adopted in movement disorders (65). Clinically, recent studies assessing the efficacy of stimulating treatments as DBS or repetitive transcranial magnetic stimulation (rTMS) at specific movement-related regions (such as globus pallidum or premotor cortices) have demonstrated some positive effects in attenuating the symptoms (66–69).

PET with perfusion radiotracers for monitoring treatments in pD and GTS is not routinely used in clinical practice but some authors, even if with a small cohort of patients, have demonstrated the feasibility and the potential role of this approach in specific setting of post-therapy assessment (see Table 1 for dystonia). Kumar and colleagues (70) studied the effects of bilateral GP DBS, by means of clinical assessment and of ^15^O-H_2_O PET, in a case of medication-refractory generalized dystonia, taking into account different stimulators settings and induced motor conditions. The authors found a positive clinical improvement after 1 year of DBS, also during active condition in terms of reaction times and randomness of movements, “due to suppression of dystonic patterned involuntary movements.” Coherently, PET investigation showed that bilateral GP DBS, during the action (moving a joystick), was reducing activity in lentiform nuclei, motor areas (primary, premotor, SMA), and also in control areas such anterior cingulate or prefrontal cortices (70). Detante et al. (7) studied the effects of internal DBS on bilateral GP by assessing regional cerebral blood flow (by means of ^15^O-H_2_O and PET) in a sample of six patients with primary generalized dystonia. Authors designed two test conditions, namely, OFF (no stimulation) and ON (unilateral DBS GPi stimulation). During OFF condition, PET data showed overactivity in DLPFC, medial and superior frontal gyrus (medFG/supFG), OFC, and thalamus. During ON condition, while stimulation was being administered to the contralateral side of the most dystonic hand (assessed during OFF condition), PET data showed a decrease of the overactivity in the same areas and also in the putamen (7). Finally, Thobois et al. studied the effects of DBS with ^15^O-H_2_O PET in GPi in five patients with tardive dystonia (induced by neuroleptics). Differences in brain perfusion induced by GPi stimulation between motor execution and rest condition confirm that the increased activity of prefrontal cortex and premotor areas can be modulated with DBS (71). Besides ^15^O-H_2_O PET, also ^18^F-FDG PET has been used in monitoring stimulation treatments (8, 72), showing a very high accuracy and reliability.

**Table 1:** **Table showing papers in literature (chronological order) adopting PET for effects assessment of stimulation treatment in dystonia**.

Reference	Dystonia	Sample	Stimulation	Region	Tracer
	type			
(70)	gD	Case study	DBS	bilGP	^15^O-H_2_O
(7)	pgD	6	DBS	bilGPi	^15^O-H_2_O
(72)	pfD	Case study	DBS + epidural cortical stimulation	GPi + pMC	^18^F-FDG
(71)	tD	5	DBS	GPi	^15^O-H_2_O
(8)	pfD	7	Epidural cortical stimulation	preMC	^18^F-FDG

*gD, generalized dystonia; pgD, primary generalized dystonia; pfD, primary focal dystonia; tD, tardive dystonia*.

**Figure 1:**
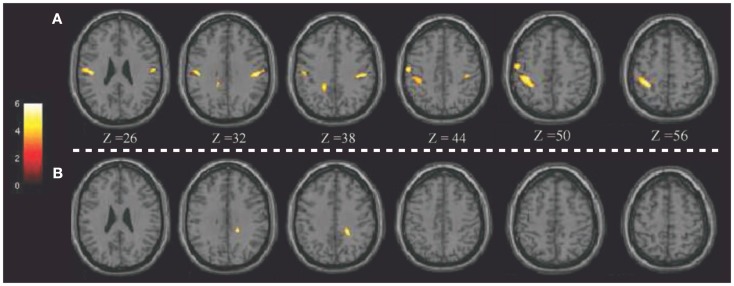
**Patients with epidural premotor cortical stimulation in primary focal dystonia**. Results of SPM8 group analysis showing increased metabolism in the patient group at **(A)** pre-surgery and **(B)** post-surgery conditions compared to normal controls. Modified from Ref. (8).

Notably, the potential of ^18^F-FDG PET in detecting regional glucose metabolism abnormalities can be improved using voxel-based statistical methods (such as SPM). This parametric approach has been applied in pD to evaluate, at a voxel-level, brain glucose metabolism changes before and after brain stimulation (8, 72). For example, Lalli et al. (8) have assessed the efficacy and safety of epidural premotor stimulation in patients with primary focal dystonia using ^18^F-FDG PET and SPM8 for statistical voxel-based analysis (VBA). In order to define regional cerebral metabolism differences, patients were compared to HCs at pre-surgery and post-surgery conditions. The authors found that the sensorimotor cortex was specifically involved in focal dystonia, with hypermetabolism (relatively to HCs) at baseline and a reduction of hyperactivity after epidural stimulation, suggesting that a positive effect of brain stimulation (Figure 1). These findings confirmed the notable role of PET to elucidate the underlying metabolic mechanisms, together with the chance to track and monitor treatments progression (8). Some years before, Romito et al. (72) used, in a case of primary fixed dystonia, ^18^F-FDG PET and SPM99 to monitor the effects of an epidural motor cortex stimulation treatment that significantly reduced the severe dystonic symptoms. This patient experienced null benefits during internal GP (GPi) stimulation, which is commonly effective in these conditions. Therefore, the authors decided to apply cortical motor areas stimulation that in 6 months, elicited notable improvements in fixed dystonia and in movements. In this study, ^18^F-FDG PET was then used to characterize the metabolic changes induced by the different treatments, revealing that GPi stimulation was inducing an increase of glucose metabolism in the sensorimotor cortex (L > R), SMA, and anterior cingulate gyri bilaterally. Conversely, motor cortex stimulation was inducing a reduction of glucose metabolism in the bilateral cerebellum. Despite the few earlier reports in literature, the author concluded that motor cortex stimulation may be an effective treatment in focal dystonia (72).

In the previous paragraphs, we have discussed how PET imaging can be useful in characterizing metabolic and neurotransmission abnormalities in GTS, suggesting that its potential application in treatment monitoring in these patients. Notwithstanding, only few reports are currently available in the literature of therapy assessment in GTS. Hopefully, future studies will further apply these techniques, given the urgency of defining effective treatments and provide appropriate safe care. Besides ^15^O-H_2_O and^18^F-FDG, PET molecular imaging may be useful too, given all the evidence of multiple neurotransmitters systems alteration in GTS.

For example, Vernaleken et al. studied, in a case study, the effect of thalamic DBS in a GTS patient by means of ^18^F-Fallypride PET (73). The aim of the authors was to clarify the mechanisms of DBS-induced modulatory effects on GTS symptoms *in vivo*. They found that this stimulation treatment exerted its effects through the modulation of the dopaminergic neurotransmission system. Given the success of this pilot study, some years later, the same group further investigated the dopamine modulation (still with ^18^F-Fallypride PET) induced by DBS in three GTS patients (9). The authors, evaluating the results, took into account all the possible confounding factors (from anesthesia for the long-PET scan duration to low-subjects number) concluding that DBS may exert its effects modulating the hyperactive dopamine transmission within the basal ganglia circuitry (9).

## Conclusion: Summary and Future Directions

The most applied PET neuroimaging approaches in movement disorders include functional and molecular imaging using ^18^F-FDG PET and neurotransmitter-specific tracers. Several studies using PET techniques, also through *ad hoc* parametric measurements or voxel-based statistical analysis (SPM) at both single-subject and group-level, have shown that regions other than the basal ganglia circuits are involved in pD and GTS. This can be used to pinpoint a breakdown of organized trans-synaptic activity, which might distinguish these disorders. Indeed, in GTS and pD, the CSTC circuit seems to play an important role in the generation of tics and dystonia. Nevertheless, the precise localization and mechanisms of these abnormalities remain disputed and are a topic of active debate and research. Finally, despite some authors have proposed the possible value of PET investigations for early and differential diagnosis of movement disorders, further researches and added knowledge on the distinct pathophysiological basis are still necessary. Despite the doubtless value of PET molecular techniques, it should always be taken into account that these results might be undermined by confounding variables, such as age at onset, comorbidities, or ongoing medical treatments.

PET studies involving larger clinical samples to investigate glucose metabolic changes and integrity of neurotransmitter systems (e.g., dopamine system with D_2_-R availability, DAT functioning and amphetamine-induced DA_REL_) and controlling for confounding variables, will surely provide further insights, particularly in the measurement of the pathophysiological abnormalities. The more these factors are evaluated and controlled, the greater the value of the results will be, especially for clinical practice.

In regards to the assessment of therapeutic effects of DBS or cortical stimulations by PET functional neuroimaging, it appears very promising and offers a wide variety of applications. PET techniques can be very useful in correctly identifying potential targets for medical and surgical therapies. These tools can clearly help in defining how the stimulations elicit their effects on the brain functioning, both at treatment site and at whole-brain circuitry level. Evaluating therapy-induced changes in metabolism or in neurotransmitters systems can be very challenging and heavily dependent on raters’ expertise. Semi-quantitative parametric approaches provide very informative data that is less affected by inter-individual observer differences. These voxel-based analyses, especially at single-subject level, have shown high accuracy in monitoring stimulation treatment effects (6, 8, 72). We strongly claim that the adoption of PET molecular and functional imaging, especially with optimized parametric approaches, is of utmost importance for monitoring of both medical and surgical therapies in pD and GTS. Future further studies in this direction are welcome, in order to evaluate the potential of this methodology in clinical practice.

## Conflict of Interest Statement

The authors declare that the research was conducted in the absence of any commercial or financial relationships that could be construed as a potential conflict of interest.
